# Effect of Immunotherapy on C-peptide Levels in Patients With Type I Diabetes Mellitus: A Systematic Review of Randomized Controlled Trials

**DOI:** 10.7759/cureus.58981

**Published:** 2024-04-25

**Authors:** Sayantan Shankar Roy, Uma Shanker P Keshri, Md Shadab Alam, Apoorva Wasnik

**Affiliations:** 1 Department of Pharmacology, Rajendra Institute of Medical Sciences, Ranchi, IND; 2 Department of Community Medicine, Rajendra Institute of Medical Sciences, Ranchi, IND

**Keywords:** immunotherapy, beta cell function, abatacept, rituximab, c-peptide level, teplizumab, type i diabetes mellitus

## Abstract

Type 1 diabetes mellitus is an autoimmune condition characterized by insulin deficiency resulting from loss of function of beta cells in the pancreas, leading to hyperglycemia and associated long-term systemic complications and even death. Immunotherapy demonstrates beta cell function-preserving potential; however, its impact on C-peptide levels, a definitive biomarker of beta cell function, and endogenous insulin secretion remain unclear. A systematic review of various immunotherapeutic interventions is hence needed for a comprehensive assessment of their effectiveness as well as identifying research gaps and influencing future research and clinical decisions.

An extensive literature search was done in PubMed, Scopus, and Cochrane Library databases using precise keywords and filters to identify relevant studies. Three independent reviewers assessed eligibility according to predetermined eligibility criteria, and data was extracted. The Cochrane risk of bias assessment tool (RoB 2.0) was used to evaluate the quality and validity of the included studies. A senior reviewer resolved discrepancies and differences of opinion between independent reviewers.

A total of 11 studies were included, with 1464 study participants. Both Phase II and III trials were included. Within the included studies, four studies assessed the anti-CD3 monoclonal antibody otelixizumab as an intervention. Another anti-CD3 monoclonal antibody, teplizumab, was assessed as an intervention in four studies, whereas two studies assessed the anti-CD20 antibody rituximab and one study assessed abatacept as its interventional drug. Otelixizumab demonstrated benefits at higher doses but was associated with adverse effects like Ebstein-Barr virus reactivation and cytomegalovirus infection, while at lower doses it failed to show a significant difference in C-peptide levels or glycosylated hemoglobin (HbA1c). Teplizumab, on the other hand, showed promise in reducing C-peptide loss and exogenous insulin requirements and was associated with adverse events such as rash, lymphopenia, urinary tract infection, and cytokine release syndrome. However, these reactions were only associated with therapy initiation, and they subsided on their own. Rituximab improved C-peptide responses, and abatacept therapy demonstrated reduced loss of C-peptide, improved C-peptide levels, and lowered HbA1c.

Teplizumab, rituximab, otelixizumab, and abatacept show potential for preserving beta cell function by reducing C-peptide loss in patients with type I diabetes mellitus. However, careful monitoring of adverse reactions, particularly viral infections and cytokine release syndrome, is necessary for the safe implementation of these therapies.

## Introduction and background

Introduction

Type 1 diabetes mellitus (T1DM) is an autoimmune condition that begins by targeting the beta cells of the pancreas, eventually destroying them and depleting endogenous insulin secretion in the body. India has around 97,700 children reported to be suffering from T1DM. At 17.93 instances per 100,000 children in Karnataka, 3.2 cases per 100,000 children, and 10.2 cases per 100,000 children in Chennai and Karnal, respectively, T1DM is becoming more common in India and is characterized by hyperglycemia, which leads to complications such as neuropathy, nephropathy, and retinopathy [1,2]. C-peptide is a widely used biomarker for assessing beta cell function in diabetes. C-peptide is produced in equimolar amounts to insulin but has a slower degradation rate, providing a more stable measure of beta cell response. In T1DM, reduced C-peptide levels result from beta-cell dysfunction, contributing to complications. Preserving beta cell function, as measured by C-peptide, improves metabolic control and reduces complications [3,4]. By significantly reducing the loss of C-peptide, immunotherapy can maintain beta cell function in patients with T1DM. Immunotherapy can halt the autoimmune attack on beta cells, preserve functional islets, and reduce C-peptide loss. Several studies have explored the relationship between immunotherapy and beta cell preservation, with some showing significant preservation of C-peptide secretion. Further research is needed to determine the optimal immunotherapy regimen for preserving beta cell function in T1DM patients [5,6]. The rationale for conducting this systematic review is to determine the effectiveness of various immunotherapy regimens for preserving beta cell function in patients with T1DM. This review synthesizes results from available randomized control trials (RCTs) and their analyses on various measures and aims to provide some valuable insights for clinicians and researchers in optimizing treatment strategies for T1DM patients.

Objective

To assess the effect of various immunotherapies on the C-peptide levels in patients with T1DM through a systematic review of available RCTs.

Primary outcome

Change in C-peptide levels from baseline in patients with T1DM measured by the area under the curve (AUC) of C-peptide levels during the mixed meal tolerance test at either two hours or four hours post-meal.

## Review

Methods

A preliminary search about T1DM and the status of immunotherapy as the treatment was conducted. Based on the preliminary searches, a PICO (Patient-Intervention-Comparator-Outcome) was devised, and a systematic literature search was conducted accordingly. Clear eligibility criteria were established.

Inclusion Criteria

Study types: Only randomized control trials (RCTs) and their analyses, which provide data on the follow-up of those studies.

Participants: Those recently diagnosed with T1DM.

Intervention: Studies evaluating immunotherapy as an intervention.

Comparator: Studies comparing immunotherapy with a control group, viz., placebo or standard therapy.

Primary outcome: Change in C-peptide levels from baseline to specified follow-up time points.

Study duration: There was no restriction on study duration.

Publication status and language: Only published studies were considered, and language was limited to English.

Exclusion Criteria

The exclusion criteria were as follows: (1) Non-randomized studies like observational studies, case reports, reviews, and editorials; (2) Animal studies; (3) Studies focusing on non-immunotherapy interventions for T1DM; and (4) Duplicate studies. 

Based on this eligibility criteria, relevant keywords and filters were applied, and searches were conducted in the databases of PubMed, Scopus, and Cochrane Library from their inception until July 1, 2023 (Table 1). 

**Table 1:** Search strategy

Search database	Search query	Result
PubMed	(("Immunotherapy"[Title/Abstract] AND "C-peptide"[Title/Abstract] AND "type 1 diabetes mellitus"[Title/Abstract]) OR "T1D"[Title/Abstract] OR "T1DM"[Title/Abstract]) AND ((excludepreprints[Filter]) AND (clinicaltrial[Filter] OR clinicaltrialphasei[Filter] OR clinicaltrialphaseii[Filter] OR clinicaltrialphaseiii[Filter] OR clinicaltrialphaseiv[Filter] OR randomizedcontrolledtrial[Filter]) AND (humans[Filter]) AND (1000/1/1:2023/7/1[pdat]) AND (english[Filter]))	937
COCHRANE	C(teplizumab):ti,ab,kw AND ("C peptide"):ti,ab,kw AND ("type I diabetes mellitus"):ti,ab,kw	2
	("rituximab"):ti,ab,kw AND ("C peptide"):ti,ab,kw AND ("type I diabetes mellitus"):ti,ab,kw	0
	(Otelixizumab):ti,ab,kw AND ("C peptide"):ti,ab,kw AND ("type I diabetes mellitus"):ti,ab,kw	0
	("abatacept"):ti,ab,kw AND ("C peptide"):ti,ab,kw AND ("type I diabetes mellitus"):ti,ab,kw	0
SCOPUS	Keywords: Teplizumab, Otelixizumab, Rituximab, Abatacept, Type I Diabetes mellitus	6

**Figure 1:**
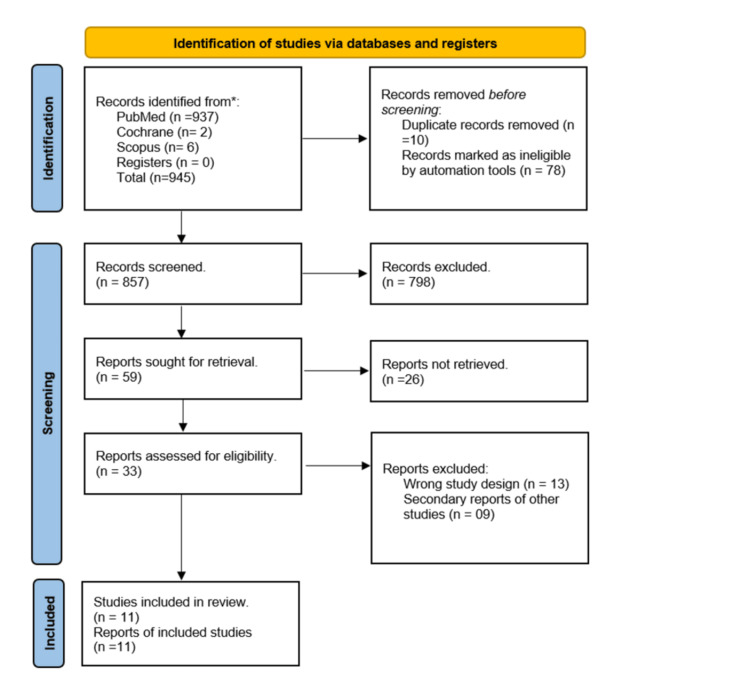
PRISMA flow diagram PRISMA: Preferred Reporting Items for Systematic Reviews and Meta-Analyses *  Results from electronic database search

Initially, two reviewers (SR and AW) screened the titles and abstracts of the studies. After initial search and screening, the systematic review was registered with PROSPERO (Review ID: CRD42023446370). Post-this full-text articles of potentially eligible studies were retrieved and assessed individually by three reviewers (SR, AW, and SA) against the eligibility. Discrepancies or differences in opinions between the reviewers were resolved through discussions and consensus. In cases where a consensus couldn’t be reached, a senior researcher (UK) was consulted to make final decisions (Figure 1).

Data Extraction

A standardized data extraction form was developed to facilitate systematic and consistent data extraction from included studies, which were coded with the author name and publication year, and in the case of the same author, name, and year of publication, they were sub-coded with lowercase alphabets, that is, a,b. Data extraction was done under several fields, viz., study design, sample size, duration of follow-up, age of study participants, intervention, comparator, dosage and dosing regimen of intervention, route of drug administration, results of primary outcomes, and adverse events reported. Each field was allocated a separate column and each study had a separate row in the form of a table in either Microsoft Excel or Microsoft Word (Microsoft Corp., Redmond, WA) (Table 2).

**Table 2:** Summary of studies included in our systematic review AUC: area under the curve, CMV: cytomegalovirus, EBV: Epstein-Barr virus, HbA1c: glycosylated hemoglobin, RCT: randomized control trial

Study ID	Study design	Sample size	Duration of follow-up	Age of study participants	Intervention	Comparator	Dosage and dosing regimen of intervention	Route of drug administration	Results of primary outcome	Adverse events reported
Aronson et al. (2014) [7]	Multicenter RCT	281	12 months	12-45 years	Otelixizumab	Placebo	Cumulative dose: 3.1 mg	Intravenous	There was no significant difference between the two groups AUC: -0.20±0.037 vs -0.22±0.025 nmol/L, p = 0.58	Papular and maculopapular rash
Demeester et al. (2015) [8]	Phase II RCT	80	18 months	12-39 years	Otelixizumab	Placebo	First four patients received a cumulative dose of 64 mg, remaining 36-48 mg	Intravenous	Significant interaction was observed between higher Insulin auto-antibody levels and C-peptide levels	EBV reactivation, CMV infection
Hagopian et al. (2013) [9]	Double-blind phase III RCT	513	2 years	8-35 years (Stratified into 8-11 years, 12-17 years, 18-35 years age groups)	Teplizumab	Placebo	209 participants received 14 full-day courses – 9,034 mcg/m2, 102 participants were 14-day low-dose group, received a total of 2,985 mcg/m2, and 99 participants were 6-day full-dose group, received 2426 mcg/m2 over 6 days	Intravenous	For 14-day full-dose group, mean change AUC = -0.136, p=0.027; for 14-day low-dose group, mean change AUC = -0.198, p=0.968; for 6-day low-dose group, mean change AUC =-0.174, p = 0.312 vs placebo = -0.191	Lymphopenia, no cytokine release events
Herold et al. (2013a) [10]	Open-label multicenter RCT	58	6 and 12 months	8-30 years	Teplizumab	Placebo	14-day course, median cumulative dose = 11.6 mg	Intravenous	Teplizumab had C-peptide levels 21.7% higher than placebo at the primary endpoint. At 12 months, teplizumab showed a significantly lower loss of C-peptide than the placebo. Participants with HbA1c<6.5% in the teplizumab group had C-peptide 56.3% higher than placebo	
Herold et al. (2013b) [11]	Open-label multicenter RCT	52	2 years	8-30 years	Teplizumab	Placebo	14-day course, Median cumulative dose = 11.6 mg	Intravenous	Teplizumab-treated group had a 75% higher mean C-peptide area under curve level at 2 years compared to control. Smaller drop in C-peptide and a higher percentage of detectable C-peptide at 24 months in the teplizumab group	
Herold et al. (2005) [12]	Phase II open-label RCT	42	2 years	7.5-30 years	Anti-CD3 Monoclonal antibody HoKT3y1(Ala-Ala)/Teplizumab	Control	First 12 subjects: D1=1.142 mcg/kg D2= 5.67 mcg/kg D3= 11.3 mcg/kg D4= 22.6 mcg/kg D5-14= 45.4 mcg/kg, Dosing modified for 13-21 subjects: D1= 460 mcg/m2 D2= 919 mcg/m2 D3= 818 mcg/m2	Intravenous	AUC of C-peptide mean± SE = 97±9.6 vs 53±7.6%, p<0.01, significant improvement in C-peptide even after 2 years	Headache, myalgia, arthralgia, urticarial rash (cytokine release syndrome-like)
Herold et al. (2011) [13]	Double-blind RCT	81	1 year	8-40 years	Rituximab	Placebo	375 mg/m2 of body surface	Intravenous	Significant differences in average responses over 12 months persisted between the groups (p=0.0013)	
Keymeulen et al. (2020) [14]	Single-blind multicenter RCT	40	2 year	16-27 years	Otelixizumab	Placebo	9 mg, 18 mg, 27 mg, 36 mg administered intravenously (4 cohorts)	Intravenous	At month 18, otelixizumab 9 mg group showed a statistically significant increase compared to placebo. Other groups had lower mean AUC C-peptide levels at month 18 compared to baseline	Cytokine release syndrome and dose-dependent and transient EBV reactivation
Orban et al. (2014) [15]	Double-masked multicenter phase II trial	112	2 years (3,6,12,18,24 months)	6-36 years	Abatacept	Placebo	10 mg/kg, max: 1000 mg/dose on D1, 14, 28, and monthly for a total of 27 infusions over 2 years	Intravenous	59% adjusted C-peptide (95% CI: 6.1%,112%) higher at 2 years with Abatacept(0.378pmol/ml) vs placebo (0.238 pmol/ml) (p=0.0029), lower HbA1c levels(p<0.02)	Infusion-related events but non-serious, no episodes of increase in infection or neutropenia
Pescovitz et al. (2009) [16]	Double-blind RCT	87	1 year	8-40 years	Rituximab	Placebo	375 mg/m2 of body surface area on D1, 8, 15, 22	Intravenous	AUC for the level of C-peptide at 2 hours in the Rituximab group was 0.56 pmol/ml (95% CI, 0.50 to 0.63) as compared to 0.47 pmol/ml(95% CI, 0.39 to 0.55). Patient who received rituximab had better levels of C-peptide at 3,6 and 12 months more pronounced at 6 and 12 months. Lower HbA1c levels over 12 months (6.76±1.24% vs 7.00±1.30%, p<0.001)	Fever, Cough, Pruritus, Grade 3 adverse reaction like Shortness of breath, Rash in only 1 patient
Ambery et al. (2016) [17]	Double-blind multicenter phase III trial	118	12 months	12-17 years	Otelixizumab	Placebo	3.1 mg over 8 days	Intravenous	Numerical difference in C-peptide AUC and p-value of 0.051 suggest a trend towards a potential effect of otelixizumab	

Risk of Bias Assessment

**Figure 2:**
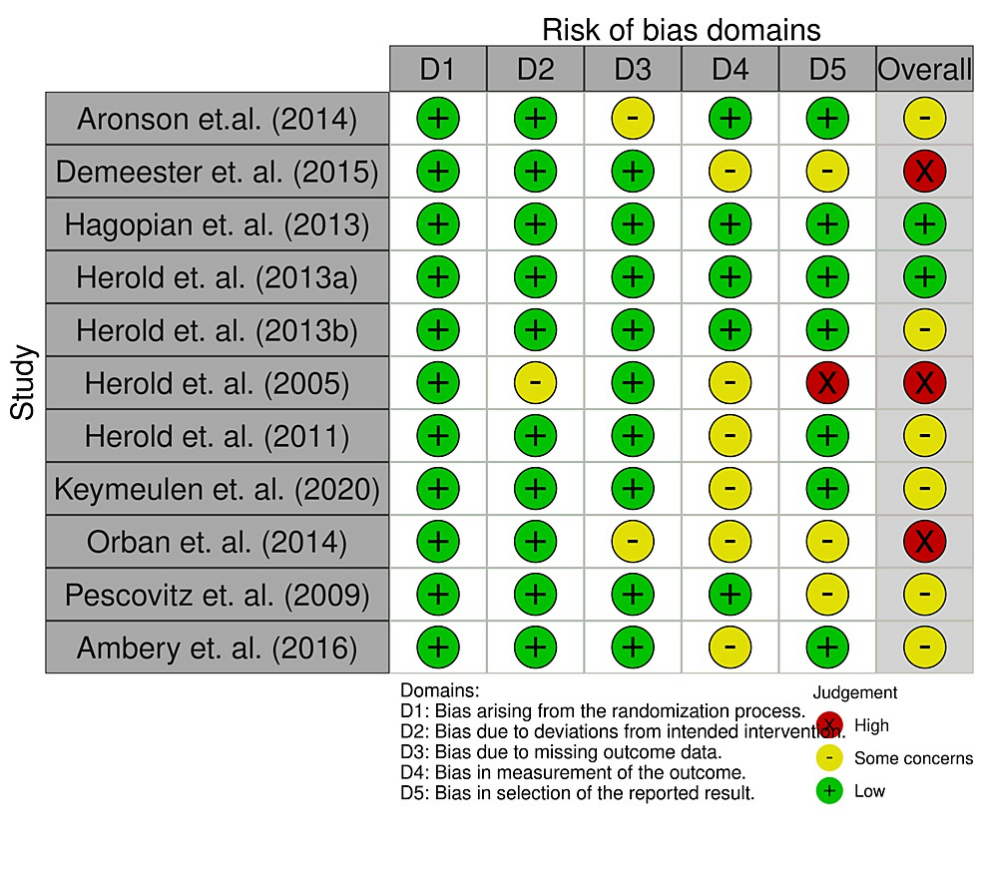
Traffic light plot of the Cochrane risk of bias assessment [7-17]

**Figure 3:**
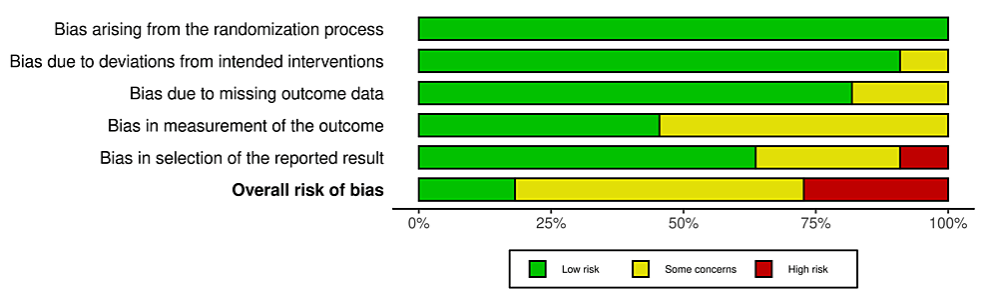
Summary plot of the Cochrane risk of bias assessment of included studies

To evaluate the risk of bias in included studies, the Cochrane risk of bias assessment tool known as RoB 2.0 was employed. The assessment covers multiple domains to evaluate potential sources of bias in the included studies, viz., random sequence generation, allocation concealment, blinding of participants and personnel, blinding of outcome assessment, incomplete outcome data, selective reporting, and other potential sources of bias. Two independent reviewers evaluated the risk of bias in each included study using the Cochrane RoB 2.0 tool. Any discrepancies or disagreements between reviewers were resolved through discussion and consensus. A Microsoft Excel spreadsheet was utilized to assign the risk of bias to each domain for all included studies. Each domain was categorized as low risk, some concerns, or high risk based on the specific criteria outlined in the Cochrane RoB 2.0 tool. Subsequently, an overall risk of bias was assigned to individual studies based on the collective assessment of all domains. The Microsoft Excel file containing the domain-specific assessments was uploaded to the robvis (Risk-of-Bias VISualization) tool [18]. A traffic light plot (Figure 2) and summary plot (Figure 3) were generated, which visually represented the distribution of the risk of bias across the included studies.

Summary of results

A total of 11 studies were included in the review, with 1464 participants. Studies assessed interventions like anti-CD3 monoclonal antibodies otelixizumab and teplizumab, anti-CD20 monoclonal antibodies rituximab, and abatacept, a co-stimulation modulator. Both phase II and III trials and their analyses were included and evaluated. Most of the studies reported results after at least 12 to 24 months of follow-up. Otelixizumab was studied over an array of dosing regimens, starting from a cumulative dose of 3.1 mg to 64 mg. Study participants who received 3.1 mg showed no significant difference in mean C-peptide levels as compared to placebo, and adverse reactions like papular and maculopapular rash were reported. Whereas in groups of higher cumulative doses such as 9 mg, 18 mg, 27 mg, and 36 mg, at 18 months of follow-up, the otelixizumab group showed a statistically significant rise in mean AUC C-peptide levels, most notably higher in the 9 mg subgroup. However, higher doses were associated with cytokine release syndrome and dose-dependent transient Ebstein-Barr virus reactivation. Teplizumab, too, was studied over a range of dose-differentiating and age-stratified subgroups. In the Phase II trial, HoKT3yl (Ala-Ala) or teplizumab showed significant improvement in C-peptide levels even after two years of follow-up. Adverse events like headache, myalgia, arthralgia, and urinary rash (like Cytokine Release Syndrome) were reported. In a phase III trial, a 14-day course of cumulative doses of around 9034 mcg/m2 exhibited the most significant mean change as compared to placebo. Even the 14-day low dose and six-day full dose showed a change but were not statistically significant. Benefits were more prominent in a younger subset of 8-11 and 12-17 years of age, as shown by subgroup analysis. Another multicenter trial studying teplizumab had C-peptide levels 21.7% higher than the placebo at the primary endpoint. Participants with HbA1c<6.5% in the teplizumab group had C-peptide levels 56.3% higher than placebo. Also, at two years of follow-up, the teplizumab-treated group had a 75% higher mean C-peptide AUC compared to the control. Dose-dependent lymphopenia, transient in nature, was reported with the use of teplizumab. Overall, teplizumab exhibited substantial clinical benefits with prolonged C-peptide enhancement and better metabolic control. Rituximab had better levels of C-peptide at three, six, and 12 months, which was much better at the sixth- and twelfth-month follow-ups. At 12 months, even HbA1c levels were lower in the rituximab group. Fever, cough, and pruritus were some of the adverse reactions reported. Quite similar were abatacept’s actions, which, at two years of follow-up in comparison to the placebo, showed higher C-peptide levels and statistically significant lower HbA1c levels.

Discussion

This systematic review assessed randomized controlled trials investigating immunotherapy effects on C-peptide levels in T1DM. Studies covered teplizumab, otelixizumab, rituximab, and abatacept, revealing teplizumab's remarkable impact: 75% C-peptide AUC increase, reduced decline, improved metabolic control, and younger age subset benefits. Otelixizumab's 9 mg dose subgroup exhibited elevated C-peptide at 18 months. Rituximab demonstrated enhanced C-peptide responses over 3-12 months, while abatacept reduced C-peptide loss over two years. Teplizumab, rituximab, and abatacept offer promising outcomes in maintaining insulin secretion, while otelixizumab's varied effects emphasize challenges in consistent benefits. The findings have significant clinical relevance, suggesting immunotherapy as a viable approach for insulin preservation. Mechanisms involve immune response modulation, reducing beta cell destruction. Teplizumab inhibits T cell activation, rituximab depletes B cells, and abatacept inhibits T lymphocyte activation to curb beta cell damage. This systematic review aligns with prior research on immunotherapy's impact on C-peptide levels in T1DM. For example, a previous study reported that teplizumab treatment reduced β-cell death at 1 year, but the differences versus placebo were not significant earlier, at six months. Another study demonstrated that though rituximab does slow down the fall in C-peptide significantly, it doesn’t quite bring about a change in the basic pathophysiology of the condition [19]. 

Limitations

Varying doses and follow-up intervals among studies affected comparability, different inclusion criteria impacted generalizability, differing sample sizes influenced statistical power, and varied response definitions affected the comparison of results. Additionally, diverse adverse event profiles impacted intervention safety. Future research must address study limitations.

## Conclusions

A series of RCTs comprehensively explored immunomodulatory interventions in recent-onset T1DM. Otelixizumab investigations showed mixed outcomes, with the 3.1 mg dosage failing to impact C-peptide or HbA1c levels over 12 months, whereas otelixizumab 9 mg led to increased C-peptide levels at 18 months, but inconsistency in glycemic control and elevated adverse events were observed. Teplizumab demonstrated remarkable efficacy, with a significant 21.7% increase in C-peptide levels and reduced loss at the primary endpoints. Prolonged effects were observed over two years, including a substantial 75% elevation in the C-peptide AUC, decreased decline, and reduced exogenous insulin use. HoKT3y1(Ala-Ala)/teplizumab showed sustained efficacy, heightened C-peptide response, prolonged clinical effects, and improved metabolic control. Rituximab interventions displayed enhanced C-peptide responses at three months, maintained significance for 12 months, and demonstrated potential therapeutic promise. Abatacept demonstrated long-term benefits, with a reduction in C-peptide loss over two years compared to placebo, implying preservation of endogenous insulin secretion. Cumulatively, these studies contribute to the evolving comprehension of immunomodulatory interventions, shaping our knowledge of their impact on C-peptide responses, glycemic control, and adverse events in recent-onset T1DM. Risk of bias assessments identified studies with lower risk, enhancing the reliability of findings. Continued research and precision-driven therapeutic tailoring remain pivotal for optimizing interventions.
